# Potency of Hexaconazole to Disrupt Endocrine Function with Sex Hormone-Binding Globulin

**DOI:** 10.3390/ijms24043882

**Published:** 2023-02-15

**Authors:** Ali Alquraini

**Affiliations:** Department of Pharmaceutical Chemistry, Faculty of Clinical Pharmacy, Al Baha University, Al Baha 65779, Saudi Arabia; aalquraini@bu.edu.sa

**Keywords:** hexaconazole, sex hormone-binding globulin, endocrine disruption

## Abstract

Hexaconazole is widely used as a fungicide for agricultural purposes. However, the endocrine-disrupting potential of hexaconazole is still under investigation. In addition, an experimental study found that hexaconazole may disrupt the normal synthesis of steroidal hormones. The potency of hexaconazole to bind with sex hormone-binding globulin (SHBG), a plasma carrier protein that binds androgens and oestrogens, is unknown. In this study, we evaluated the efficacy of hexaconazole to bind with SHBG by molecular interaction, a molecular dynamics method. In addition, principal component analysis was performed to understand the dynamical behaviour of hexaconazole with SHBG in comparison with dihydrotestosterone and aminoglutethimide. The binding scores of hexaconazole, dihydrotestosterone, and aminoglutethimide with SHBG were found to be −7.12 kcal/mol, −11.41 kcal/mol, and −6.84 kcal/mol, respectively. With respect to stable molecular interaction, hexaconazole showed similar molecular dynamics patterns of root mean square deviation (RMSD), root mean square fluctuation (RMSF), radius of gyration (Rg), and hydrogen bonding. The solvent surface area (SASA) and principal component analysis (PCA) of hexaconazole exhibit similar patterns in comparison with dihydrotestosterone and aminoglutethimide. These results show that hexaconazole has a stable molecular interaction with SHBG, which may acquire the active site of the native ligand, resulting in significant endocrine disruption during agricultural work.

## 1. Introduction

Hexaconazole is a triazole fungicide used to control various types of fungal infections in various fruits and vegetables [1]. One of the most serious types of chemical pollution in the modern period is exposure to pesticides. Various research studies have revealed that pesticides and/or their metabolites may interfere with hormone receptors as hormone agonists or antagonists, acting as endocrine-disrupting chemicals (EDCs) [2]. Hexaconazole is a carcinogen as per the categorization of the U.S. EPA (Category C) (U.S. EPA, 2006). Hexaconazole has a high level of stability and its rate of degradation in field soil is approximately 225 days; because of hexaconazole’s stable nature, there could be a risk of exposure, which may lead to an unknown etiological factor of abnormal haemostasis of various hormonal syntheses. However, thyroid endocrine disruption in zebrafish due to exposure to hexaconazole has been reported by Yu L et al., 2013 [3]. In addition, hexaconazole efficacy in disrupting CYP3A4 has been previously reported [4,5].

In recent years, triazole fungicides have received a lot of attention due to reports that they may damage the endocrine system, including steroid hormones [6,7]. There are numerous potential mechanisms by which these fungicides could cause androgenic disruption [7]. Although many different pathways of triazole-induced endocrine-disrupting actions, such as oestrogen receptor and thyroid hormone receptor, have received attention, there is still a knowledge vacuum in these areas.

However, sex hormone-binding globulin (SHBG) has also been reported as a potential target for endocrine disrupters or pesticides [8]. SHBG is a glycoprotein that binds to oestrogens and androgens and when SHBG is synthesized in the sertoli cells, it is recognized as an androgen-binding protein (ABP) [9]. It has been reported that the ABP binds to testosterone and dihydrotestosterone, which may decrease their lipophilicity. These findings reflect the importance of SHBG in the normal homeostasis of androgen and oestrogen [9]. In addition, because of its high ligand-binding affinity, SHBG serves as a key carrier protein for steroids in the blood, and variations in SHBG levels may impact the transportation of steroid molecules, such as dihydrotestosterone and testosterone, by target tissues. However, it appears that there are no molecular modelling studies of hexaconazole with human SHBG. The aim of the current work is to examine the structural-binding properties of hexaconazole with SHBG by using molecular docking molecular dynamics methods.

## 2. Results

### 2.1. SHBG Molecular Interaction Pattern

**Figure 1:**
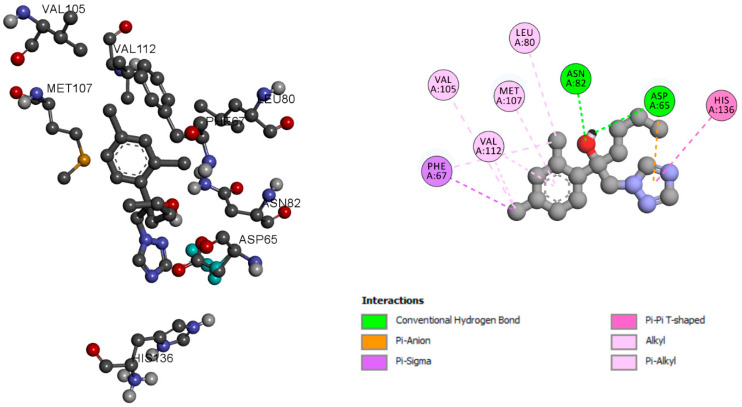
SHBG molecular interaction pattern with hexaconazole.

**Figure 2:**
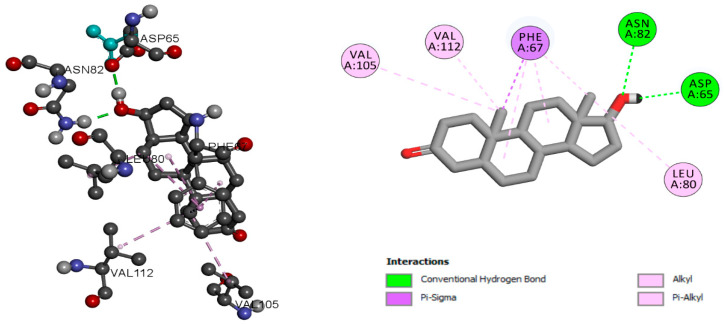
SHBG molecular interaction pattern with dihydrotestosterone.

Hexaconazole has a binding score of −7.12 kcal/mol and alkyl and Pi-Alkyl molecular interaction with residues VAL 105, MET 107, LEU 80, and VEL 112. Two residues, ASN82 and ASP 65, were associated with conventional hydrogen bond interaction. The residue PHE 67 was associated with Pi-sigma interaction, as shown in Figure 1. Dihydrotestosterone is an endogenous androgen sex steroid and hormone. Dihydrotestosterone plays an important role in sexual differentiation of the male genitalia during embryogenesis. To understand the pattern of molecular interaction, dihydrotestosterone was used, which is a native ligand of SHBG (ID2S). Dihydrotestosterone with SHBG was found to have a binding score of −11.41 kcal/mol, and has molecular interaction with residues VAL 105, VAL 112, and LEU 80. It was stabilized with alkyl and Pi-Alkyl bonds, residues ASN82 and ASP 65 were stabilized with conventional hydrogen bonds, and residue PHE 67 was stabilized with Pi-sigma interaction, as shown in Figure 2.

**Figure 3:**
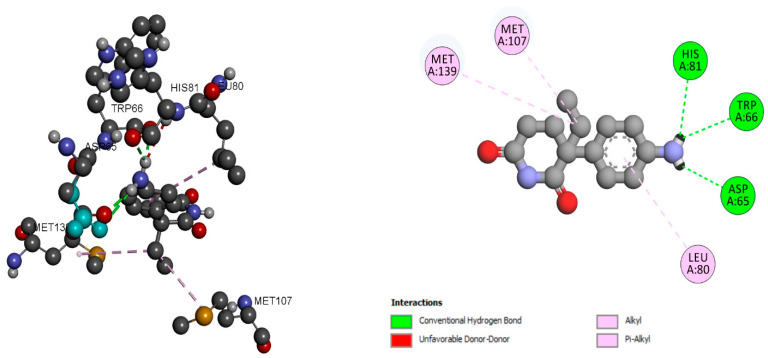
SHBG molecular interaction pattern with aminoglutethimide.

Aminoglutethimide may significantly suppress dihydrotestosterone, which is a natural ligand for SHBG. Aminoglutethimide has a binding score of −6.84 kcal/mol and has molecular interaction with residues MET 139, MET 107, LEU 80, and HIS 81, as well as with TRP 66 and ASP 66 shown as alkyl and Pi-Alkyl and hydrogen bonds, respectively, as shown in Figure 3. The details of the molecular interactions are given in Table 1. Figures S1–S4 provide the information about the active sites of the protein. In addition, the molecular interaction analysis of hexaconazole-similar azole fungicide compounds with 1D2S is given in Supplementary File S1 (Table S1, Figures S1–S10, Image S1).

### 2.2. Trajectories Analyses of MD Simulation

Analyses of simulation convergence were conducted in terms of the root mean square deviation (RMSD), the radius of gyration (RG), the root mean square fluctuation (RMSF), and the solvent-accessible surface area (SASA), which were calculated using g_rmsf, g_gyrate, and g_hbond plugins of GROMACS, respectively [5,10,11,12,13]. In addition, PCA was also calculated from the trajectories. 

**Figure 4:**
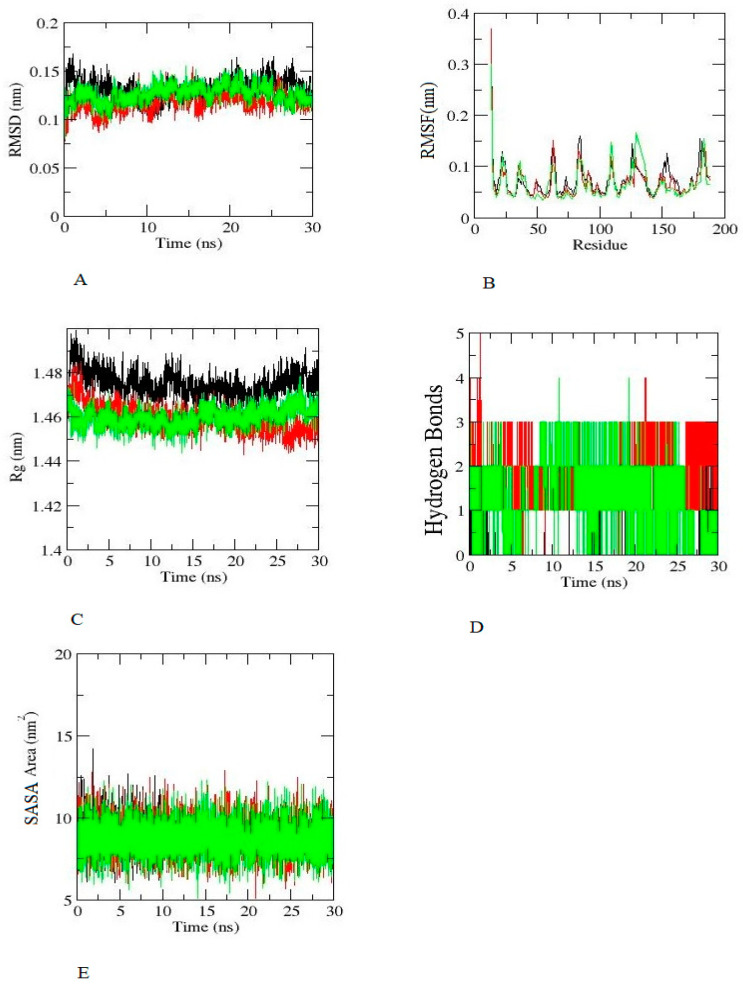
Molecular dynamics simulation of SHBG with hexaconazole (black colour), dihydrotestosterone (green colour), and aminoglutethimide (red colour). (**A**) RMSD and time (**B**) RMSF and residue (**C**) Rg and time (**D**) Hydrogen bond and time (**E**) SASA Area and time.

**Figure 5:**
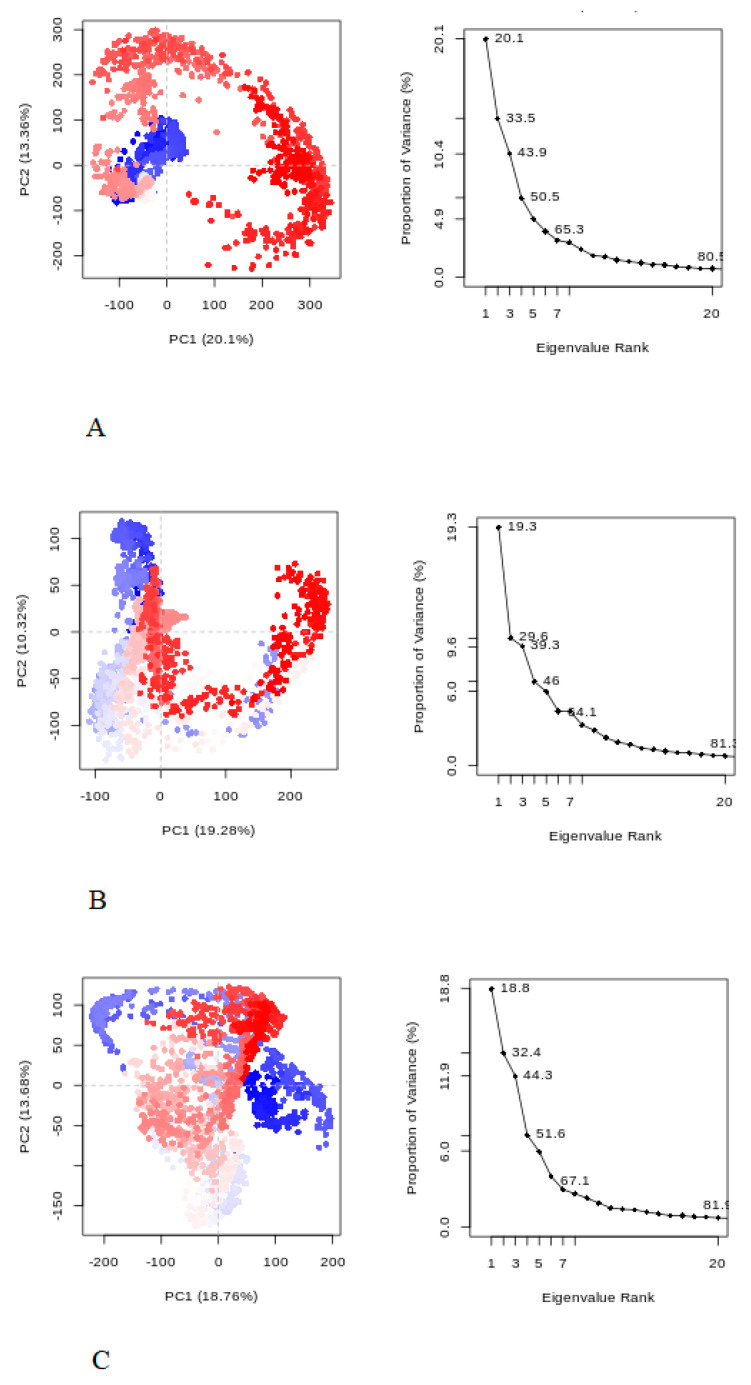
Principal component analysis (**A**) hexaconazole, (**B**) dihydrotestosterone, and (**C**) aminoglutethimide.

From the results, it was observed that each complex was well equilibrated during RMSD (Figure 4A) and displayed small variance during MD simulation. The root mean square fluctuation investigates the flexibility among the residues. Figure 4B depicts that the RMSF of hexaconazole has restricted movement similar to aminoglutethimide and dihydrotestosterone. The radius of gyration (Rg) was assessed to understand conformational changes throughout the MD simulation, and the hexaconazole complex represents a similar pattern of conformational changes (Figure 4C). In addition, hexaconazole is able to form a hydrogen bond (Figure 4D); a comparison of hydrogen bonds formed in 10 ns and 30 ns is given in Figure S3. The SASA stands for the solvent–enzyme–ligand complex interaction during MD simulation; hexaconazole SASA values of 8–12 nm^2^ were similar in comparison with aminoglutethimide and dihydrotestosterone (Figure 4E). The PCA of each complex is represented in Figure 5. The PCA predicts the significant motions and binding abilities of the substrate in the complexes. The eigenvectors successfully captured 89% of the motion in the trajectory. In addition, principal component analysis was performed to predict the significant motions in the trajectory. PCA predicted the binding of the substrate in the complexes. The eigenvectors successfully captured 80% of the motion for hexaconazole (Figure 5A), 81% dihydrotestosterone (Figure 5B), and again 81% for aminoglutethimide (Figure 5C). The graph, which shows variation in the conformational distribution, portrays each dot as one complex confirmation.

### 2.3. Molecular Mechanics of the Poisson–Boltzmann Surface Area (MM-PBSA) 

The quantitative evaluation of the substrate molecule interaction mechanism for the entire assessment is provided by MM-PBSA. The binding free energy (ΔG), van der Waals energy, and electrostatic energy with hexaconazole in comparison with dihydrotestosterone and aminoglutethimide were calculated [10]. The details are given in Table 2.

### 2.4. ADME and Toxicity Analyses

As per the U.S. EPA, hexaconazole is a Category C carcinogen. However, we explored various attributes of its toxicological profile. The hexaconazole does not violate the Lipinski rule and various attributes of its ADME profile mimic dihydrotestosterone and aminoglutethimide. The details are given in Table 3. 

## 3. Discussion

Hexaconazole is mainly used in agriculture for the control of various diseases, specifically rice sheath blight disease. However, hexaconazole has good potency for bioaccumulation, and it has also been found that hexaconazole has good potential to disrupt activities of antioxidant enzymes and malonaldehyde, cytochrome P450, and 8-hydroxy-2-deoxyguanosine in earthworms (*Eisenia fetida*). In addition, the transcriptome sequencing results show that hexaconazole significantly modulates steroid biosynthesis, arachidonic acid metabolism, and cell cycle processes, which may lead to abnormal biological function activities in earthworms *Eisenia fetida* [14]. 

Similarly, as per the study on zebrafish, hexaconazole has the ability to disrupt antioxidant enzymes such as superoxide dismutase (SOD), catalase (CAT), glutathione peroxidase (GPx), and glutathione (GSH). Moreover, hexaconazole may also induce apoptosis via caspases in adult zebrafish [3]. In addition, it has been found that hexaconazole may decrease leydig cell testosterone secretion and androgen receptor activation [2]. However, the local bioavailability of androgen or progesterone may be influenced by SHBG; because SHBG binds to testosterone and dihydrotestosterone, these hormones are made less lipophilic and become concentrated within the luminal fluid of the seminiferous tubules. The higher levels of testosterone and dihydrotestosterone empower spermatogenesis in the seminiferous tubules and sperm maturation in the epididymis. Therefore, SHBG plays an important role in the normal homeostasis of reproductive hormones. 

In the hexaconazole, hydrogens at position 2 of the hexyl chain are replaced by hydroxy and 2,4-dichlorophenyl groups; it also has chelator properties [15,16]. A ligand with chelator properties may have two or more separate binding sites, which could be the reason for the good binding score of hexaconazole, and it forms a hydrogen bond with the residues of active sites ASN 82 and ASP 65, as shown in Supplementary File S1. Other azole fungicides do not show potential interaction with SHBG, reflecting a specific geometrical feature of hexaconazole. For example, azole fungicide propiconazole substituted the dioxolan group for the normal alkyl group that bonded with the hydrogen backbone and is not able to bind with the active site, as shown in Supplementary File S1. At an atomistic level, we performed deep molecular analysis to understand the behaviour of hexaconazole to bind with SHBG in comparison with native ligand dihydrotestosterone and inhibitor aminoglutethimide. Our analyses reveal that hexaconazole has good binding potential with a binding score of −7.12 kcal/mol. In addition, as per the study of Hammond et al., 2017 [17], dihydrotestosterone has a very potent affinity to bind with SHBG. Our study corroborates this finding with a binding score of −11.41 kcal/mol. However, the binding score with inhibitor aminoglutethimide was found to be −6.84 kcal/mol; these results reflect that hexaconazole has enough potential to modulate the normal homeostasis of SHBG. The common interaction residues with SHBG hexaconazole, dihydrotestosterone, and aminoglutethimide were ASN 82 and ASP 65, which were stabilized by hydrogen bonds; VAL 105 and VAL 112 were stabilized as alkyl and Pi-Alkyl bonds; residue PHE 67 was stabilized by Pi sigma bond; residue ASP 65 was stabilized by hydrogen bonds; and residue MET 107 was stabilized by alkyl and Pi-Alkyl bond. These findings represent that hexaconazole endocrine disruption potential has a large spectrum. For example, as per the study on zebrafish, hexaconazole may disrupt thyroid function [3]. In addition, to understand the stability of hexaconazole interaction, we conducted an MD simulation study and it reflected that hexaconazole has a stable molecular interaction similar to the native ligand dihydrotestosterone and inhibitor aminoglutethimide. Hexaconazole may acquire an active site of SHBG and may disrupt the normal function of SHBG, leading to endocrine disruption. These findings may be helpful in under-standing the potential of hexaconazole to disrupt the normal function of SHBG at the atomistic level.; It has been also endorsed by experimental studies that hexaconazole has the potency to disrupt normal endocrine or reproductive hormone homeostasis by causing abnormal hormonal synthesis. Furthermore, SASA and PCA reflect the surface area of a protein that interacts with its solvent molecules and the total expansion of a protein during MD simulation, respectively [10]. Our results reveal that SHBG and the hexaconazole complex have SASA values almost similar to SHBG native ligand dihydrotestosterone and inhibitor aminoglutethimide. To identify the endocrine-disrupting effects of xenobiotics, the MD technique is successfully applied [3]. Furthermore, the binding free energy was calculated from the MD trajectories as given in Table 2, which validated the results of the molecular docking and MD simulation. In addition, PCA measure a protein’s large-scale average motion, displaying the structures underlying the atomic fluctuations and eigenvalues, which is a total motility measurement in the system [15]. The system’s total motility is quantified by eigenvalues. Comparisons of the protein’s flexibility were made under certain circumstances. The eigenvalues for hexaconazole were close to the native ligand dihydrotestosterone and the aminoglutethimide inhibitor during MD simulation. However, it was found that SHBG and the hexaconazole complex occupied significant space with a stable cluster denoted to express that the complex is highly stable. Additionally, the ADME study showed that hexaconazole’s topological polar surface area, which is the total surface area of all its polar atoms or molecules, is close to that of its native ligand dihydrotestosterone and the aminoglutethimide.

## 4. Materials and Methods

### 4.1. Preparation of the Structure and Molecular Docking

The X-ray crystal structure of SHBG (1D2S) was acquired from Protein Data Bank (PDB). The native ligands are dihydrotestosterone PUBChem Id 10635 and aminoglutethimide PUBChem Id 2145, which were used to understand the interaction pattern of hexaconazole 66461.

For molecular docking analyses, hexaconazole was docked with SHBG using the program AutoDock version 4.2 (San Diego, CA, USA) [18]. For the precise molecular interaction analyses, all crystallographic water was removed from SHBG (1D2S) protein and energy was minimized by Swiss PDBViewer, and the active site was obtained from PDBSUM. In addition, ligand energy was minimized by chimera (dihydrotestosterone PUBChem Id 10635, aminoglutethimide PUBChem Id 2145, and hexaconazole 66461). Before docking was run with AutoDock version 4.2, the structure of the target was optimized by Kollman with combined charges and solvation parameters. After this, hydrogen was added to SHBG in ideal geometry, and torsions were fixed. In addition, the protein’s van der Waals well depth was assigned, and the files were saved in PDBQT format. For the generation of grid parameters, AutoDock tools were used, and grid parameter files (GPFs) and docking parameter files (DPFs) were generated. For ligand and protein interaction analyses, the Lamarckian genetic algorithm [18] was applied. A total of 50 different poses were used to obtain the binding score [19]. The best complex was taken for molecular dynamics (MD) studies on the basis of a high binding score. For the visualization of the complex, Discovery Studio 16, molecular visualization software (Biovia, 2019) was used. In addition, molecular interaction analysis of hexaconazole-similar azole fungicide compounds with 1D2S was performed to understand the chemical feature of hexaconazole.

### 4.2. Molecular Dynamics (MD) Simulation Analyses

The structural and dynamics changes in the complex of SHBG and the respective ligand with the dihydrotestosterone, aminoglutethimide, and hexaconazole were assessed. The duration of the MD simulation was 30 ns in triplicate using GROMACS 2021 software, (Groningen, The Netherlands) and the Charmm 27 force field was applied to all atoms [20]. SHBG and the respective complex were solvated by simple point charge (SPC) water molecules. In addition, counter ions (Cl or Na) to neutralize the protein [21] were applied. Furthermore, van der Waals contacts between the atoms were eliminated by energy minimization. The complex was equilibrated in two phases. The next step was a constant number of particles, volume, and temperature (NVT) ensemble with endothermic and exothermic processes, which was exchanged with the thermostat, followed by a constant number of particles, pressure, and temperature (NPT) ensemble at 300 K with constant pressure. The linear constraint solver (LINCS) algorithm was then used to constrain the covalent bonding approach. Finally, 30 ns of MD was run to examine the stability of each system. MD was performed 3 times to verify the MD findings [10,11,12,13]. The details related to the set-up of MD simulations are given in Table 4.

### 4.3. Analyses of Trajectory

Using the GROMACS g rmsd plugin, the simulation results were represented in terms of the root mean square deviation (RMSD), the root mean square fluctuation (RMSF), the radius of gyration (RG), intermolecular hydrogen bonds [17], and the solvent-accessible surface area (SASA).

### 4.4. Principal Component Analysis (PCA) 

The principal component analysis method was used for the analysis of dynamical behaviour in conformational and substrate binding prediction in the trajectory for the main chain with a 30 ns time frame of the trajectory. The analysis was performed by the Galaxy server as per the protocol [15,22].

### 4.5. Molecular Mechanics of the Poisson–Boltzmann Surface Area (MM-PBSA) 

Hexaconazole, dihydrotestosterone, and aminoglutethimide binding free energy analyses for SHBG were calculated. The binding free energy was calculated as per the protocol by MM-PBSA. The van der Waals energy and electrostatic energy of hexaconazole were calculated in comparison with dihydrotestosterone and aminoglutethimide. The following methods were used to calculate the binding free energy:

ΔG bind = G complex − (G Protein + G Ligand)

ΔG represents the binding free energy of the ligand (hexaconazole, dihydrotestosterone, and aminoglutethimide) with SHBG, G protein represents the SHBG binding energy, and G represents the ligand [10].

### 4.6. ADME and Toxicity Analyses

The ADMET acronym stands for absorption, distribution, metabolism, elimination, and toxicity, and it provides important details about toxicokinetic properties. Hexaconazole’s ADME and toxicity characteristics were predicted by AdmetSAR 2.0, and details are given in Table 4.

## 5. Conclusions

In this study, we looked into the molecular interactions between hexaconazole and SHBG receptors. The findings show that hexaconazole has the potential to form stable molecular interactions with SHBG and its exposure may not allow native ligands to bind with SHBG, which may lead to endocrine disruption. More clinical and experimental studies are required to establish the safety aspects of hexaconazole on human health, especially exposure during agricultural work.

## Figures and Tables

**Table 1:** Molecular interaction analyses of SHBG with different ligands.

Ligands	Amino Acid Residues Involved in Hydrogen Bonds	Docking Final Intermolecular Energy (ΔG) = vdW + Hbond + Desolv Energy (kcal/mol)	Inhibition Constant (Ki)	Protein
Hexaconazole	ASN 82 ASP 65	−7.12	6.03 µM	1D2S
Dihydrotestosterone	ASN 82 ASP 65	−11.41	4.33 nM	
Aminoglutethimide	HIS 81 TRP 66 ASP 65	−6.84	9.86 µM	

**Table 2:** MM-PBSA of SHBG.

Ligand–Hormone Complex	Binding Free Energy (ΔG) (kj/mol)	Van der Waals Energy (kj/mol)	Electrostatic Energy (kj/mol)
Hexaconazole + SHBG	−26.07 ± 1.5	−40.33 ± 0.03	−2.37 ± 0.9
Dihydrotestosterone + SHBG	−30.96 ± 9.96	−43.13 ± 0.02	−23.45 ± 0.1
Aminoglutethimide + SHBG	−22.66 ± 0.59	−31.84 ± 0.08	−8.90 ± 0.17

**Table 3:** ADME and toxicity analyses.

SN	Paracetamol	Hexaconazole	Dihydrotestosterone	Aminoglutethimide
1.	Hepatotoxicty	Yes	Yes	Yes
2.	Thyroid receptors binding capacity	Yes	Yes	Yes
3.	Plasma protein binding	Yes	Yes	Yes
4.	Molecular weight	314.21 g/mol	290.45 g/mol	232.28 g/mol
5.	GI absorption	High	High	High
6.	Log Kp (skin permeation)	−5.45 cm/s	−5.45 cm/s	−6.85 cm/s
7.	Lipinski violation	No	No	No
8.	Topological surface area	50.94 A^2^	37.30 A^2^	72.19 A^2^
9.	Acute oral toxicity	Yes	Yes	Yes
10.	Respiratory toxicity	Yes	Yes	Yes

**Table 4:** Detailed set-up of simulations.

System	Protein Residues	Water	Counter Ions	Total Atoms
Hexaconazole + SHBG	2635	19,377	5 Na^+^	22,060
Dihydrotestosterone + SHBG	2635	19,377	5 Na^+^	22,068
Aminoglutethimide + SHBG	2635	19,368	5 Na^+^	22,039

## Data Availability

Not applicable.

## Supplementary Materials

The following supporting information can be downloaded at: https://www.mdpi.com/article/10.3390/ijms24043882/s1.
