# Supplementary material for: Potency of Hexaconazole to Disrupt Endocrine Function with Sex Hormone-Binding Globulin
Source: Int J Mol Sci. 2023 Feb 15;24(4):3882. doi: 10.3390/ijms24043882 (PMC9964258; doi:10.3390/ijms24043882)
Supplement: Supplementary file 1 [file ijms-24-03882-s001.zip › ijms-2123456-supplementary.pdf]

Supplementary Table S1. Molecular interaction analyses of SHBG with additional molecules.

Ligands and PubChem ID	Category of molecules	Amino acid residues involved in Hydrogen Bonds	Docking Final Intermolecular Energy (Δ G)= vdW + Hbond + desolv Energy (*kcal/mol*)	Inhibition Constant (Ki)	Protein
Hexaconazole	Azole	ASN 82<br>ASP 65	-7.12	6.03 μM	1D2S
Cyproconazole 86132	Azole	ASN 82	-6.94	8.24 μM	
Etaconazole 91673	Azole	No hydrogen bon	-7.6	2.66 μM	
Myclobutanil 6336	Azole	No hydrogen bon	-7.01	7.33 μM	
Penconazole 91693	Azole	ASN 82	-7.28	4.61 μM	
Propiconazole 43234	Azole	MET 107	-7.91	1.59 μM	

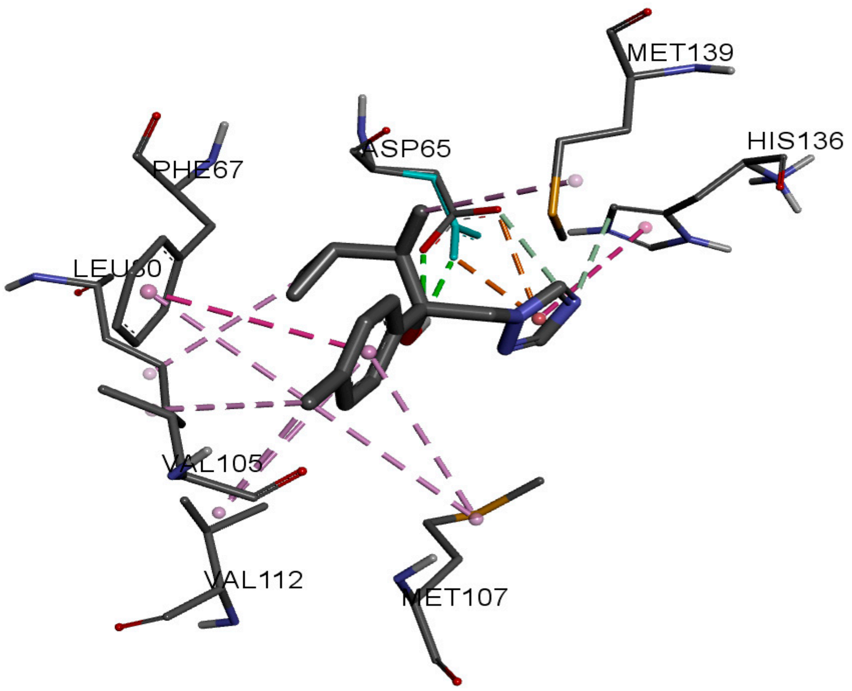

A

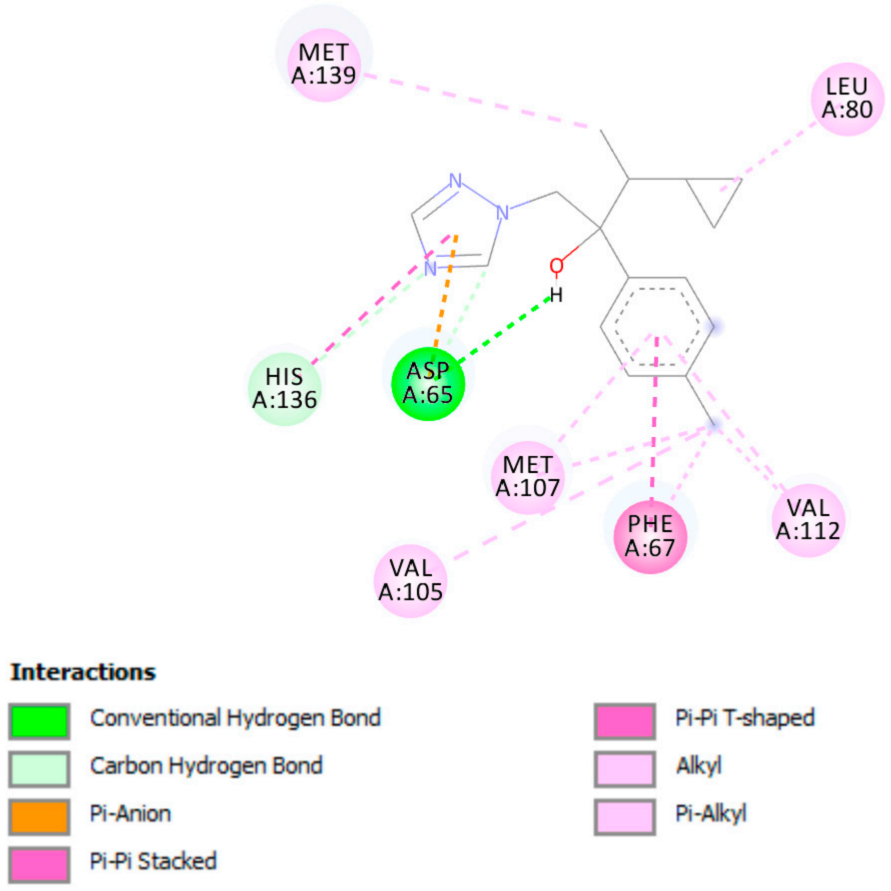

B

Figure S1. SHBG molecular interaction pattern with Cyproconazole (**A**) Molecular interaction; (**B**) 2D representation.

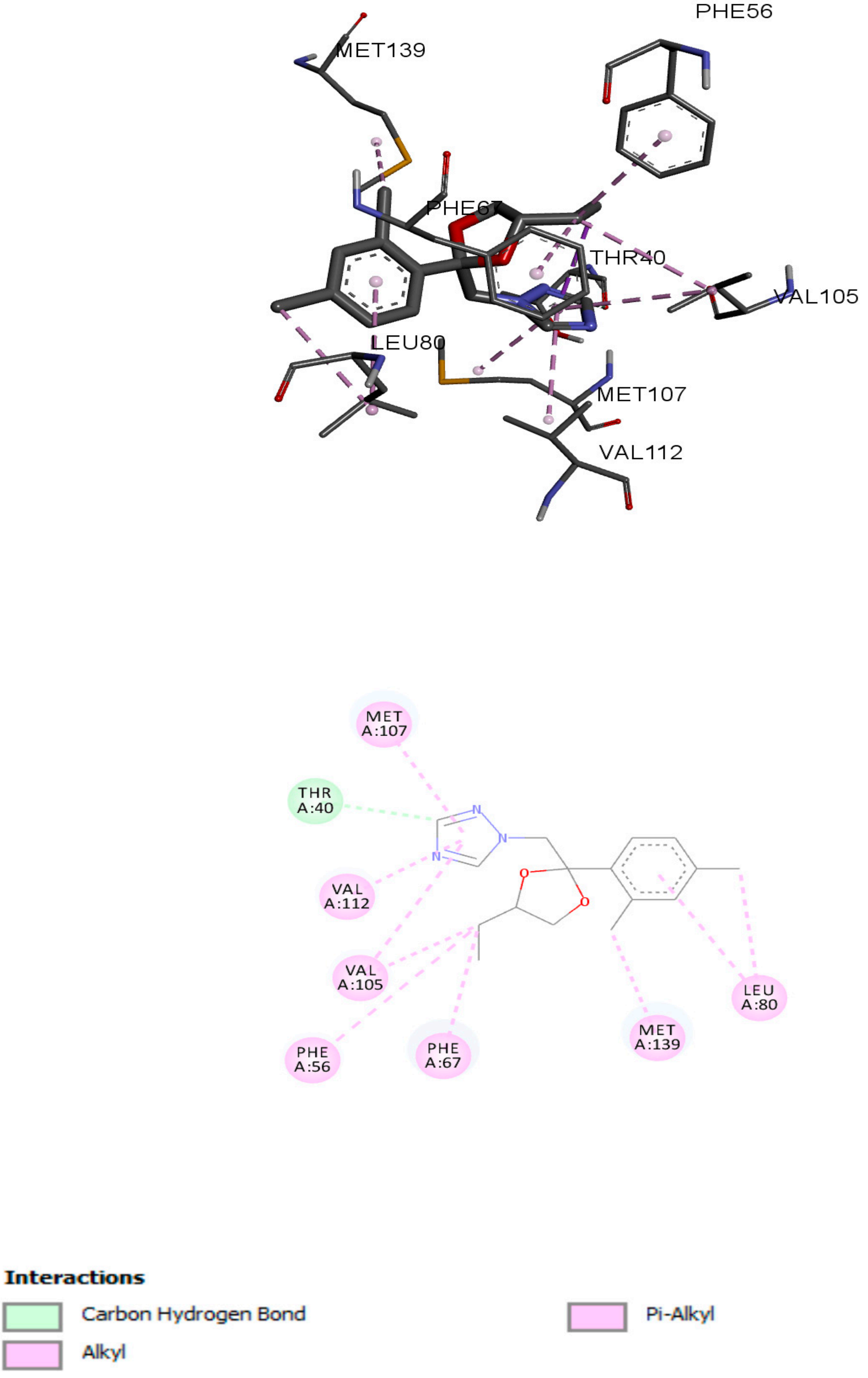

A

B

Figure S2. SHBG molecular interaction pattern with Etaconazole (**A**) Molecular interaction; (**B**) 2D representation.

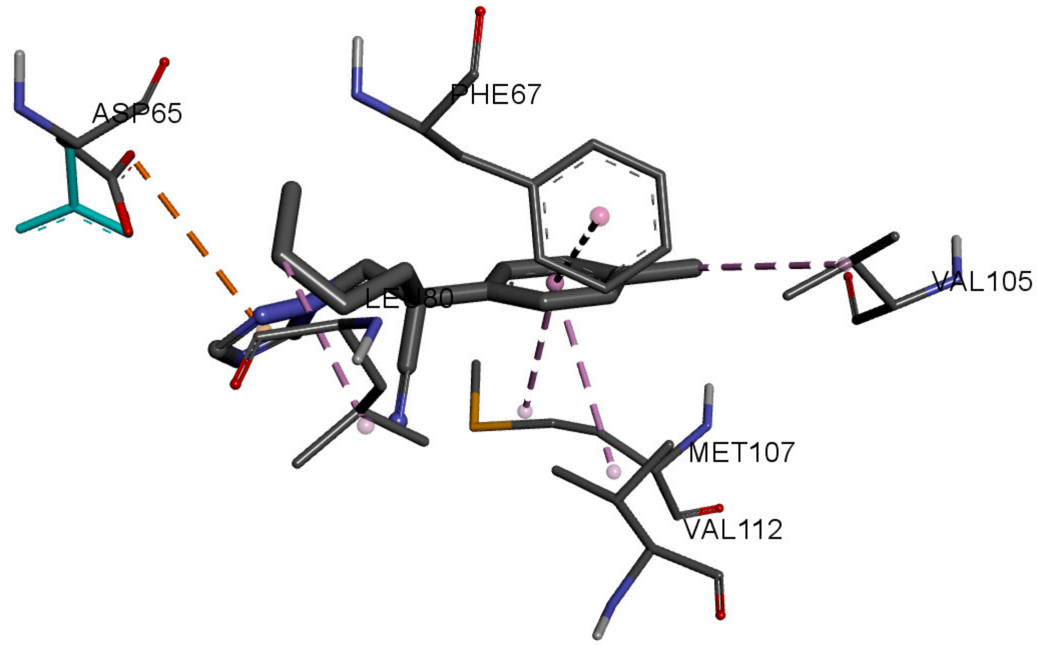

A

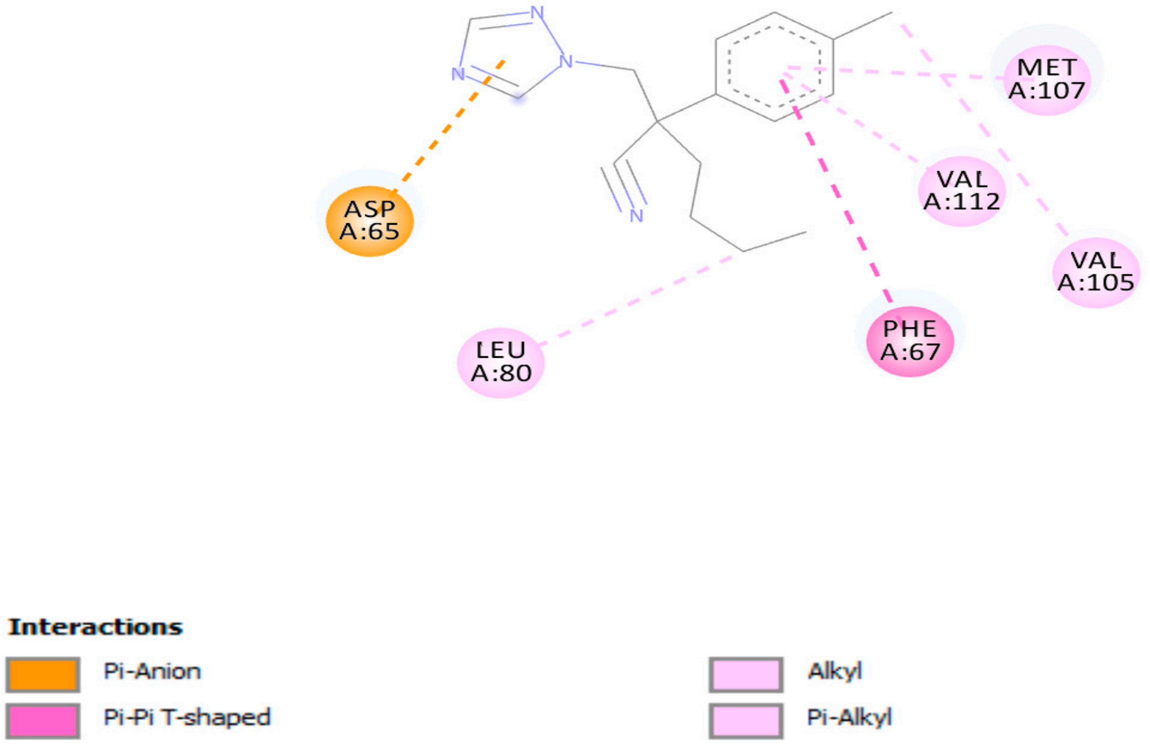

B

Figure S3. SHBG molecular interaction pattern with Myclobutanil (**A**) Molecular interaction; (**B**) 2D representation.

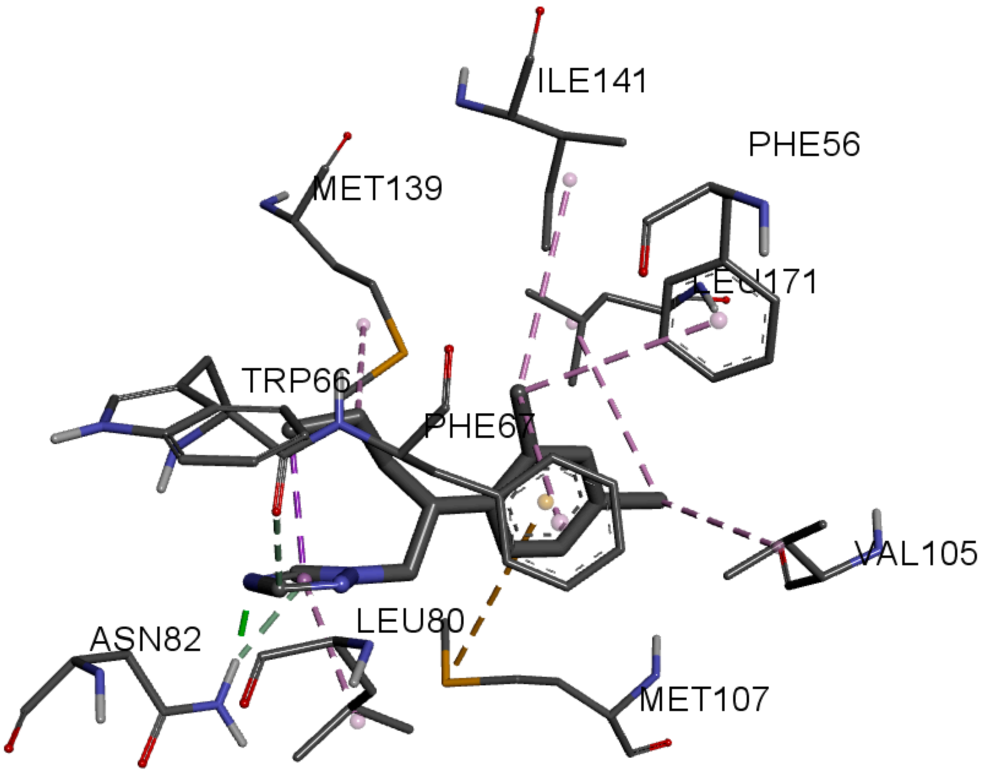

A

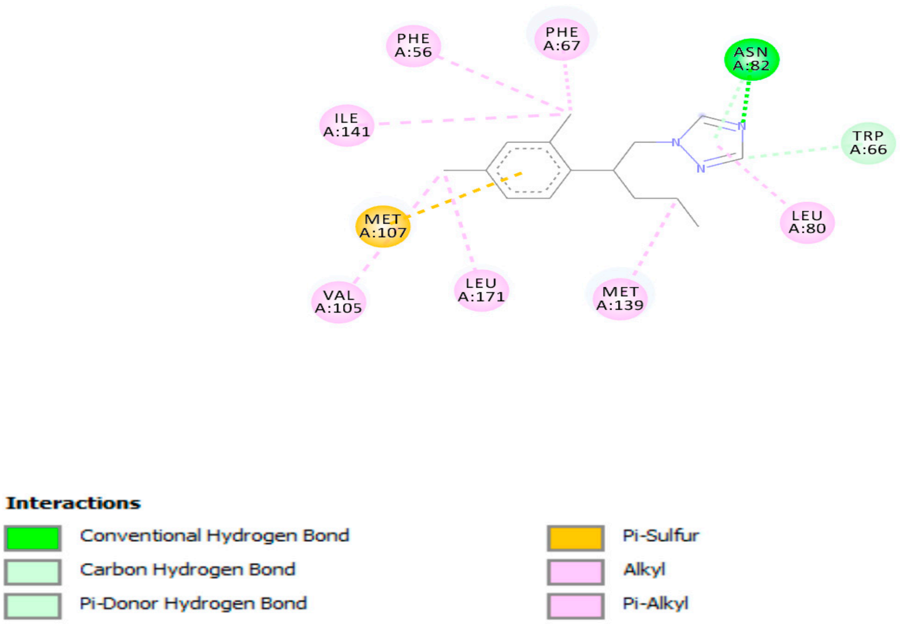

B

Figure S4. SHBG molecular interaction pattern with Penconazole (**A**) Molecular interaction; (**B**) 2D representation.

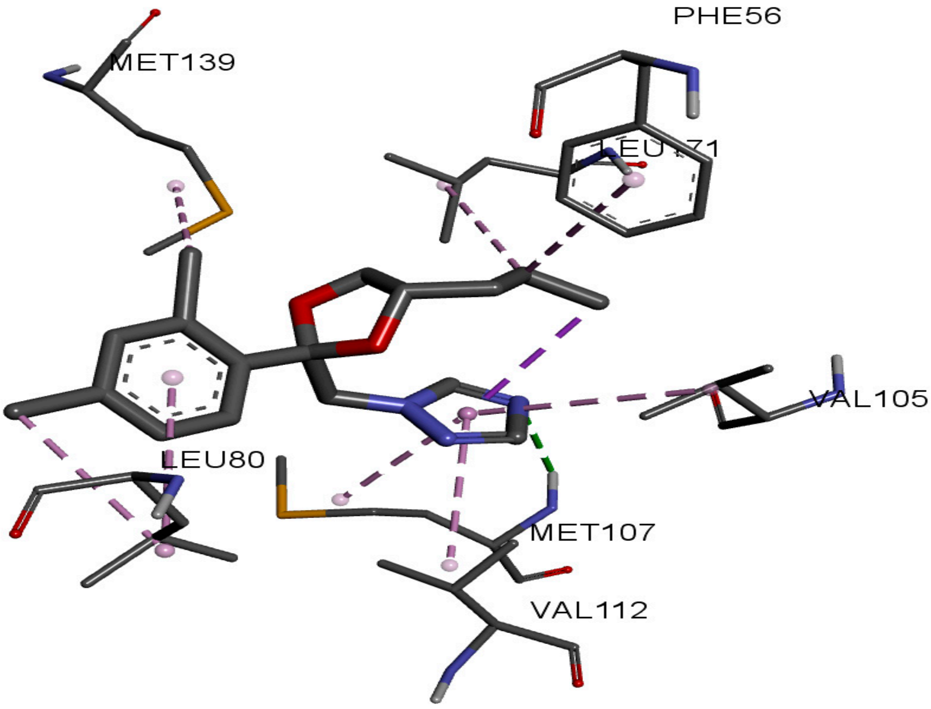

A

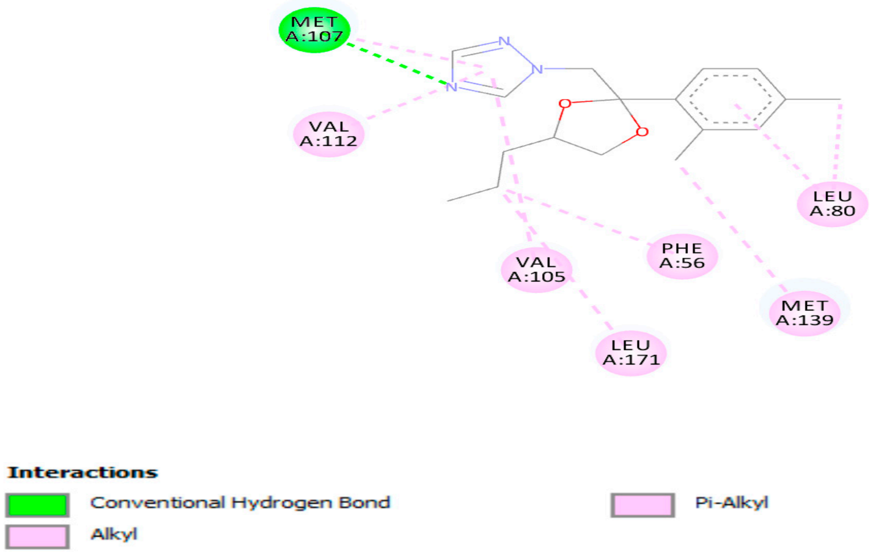

B

Figure S5. SHBG molecular interaction pattern with Propiconazole (**A**) Molecular interaction; (**B**) 2D representation.

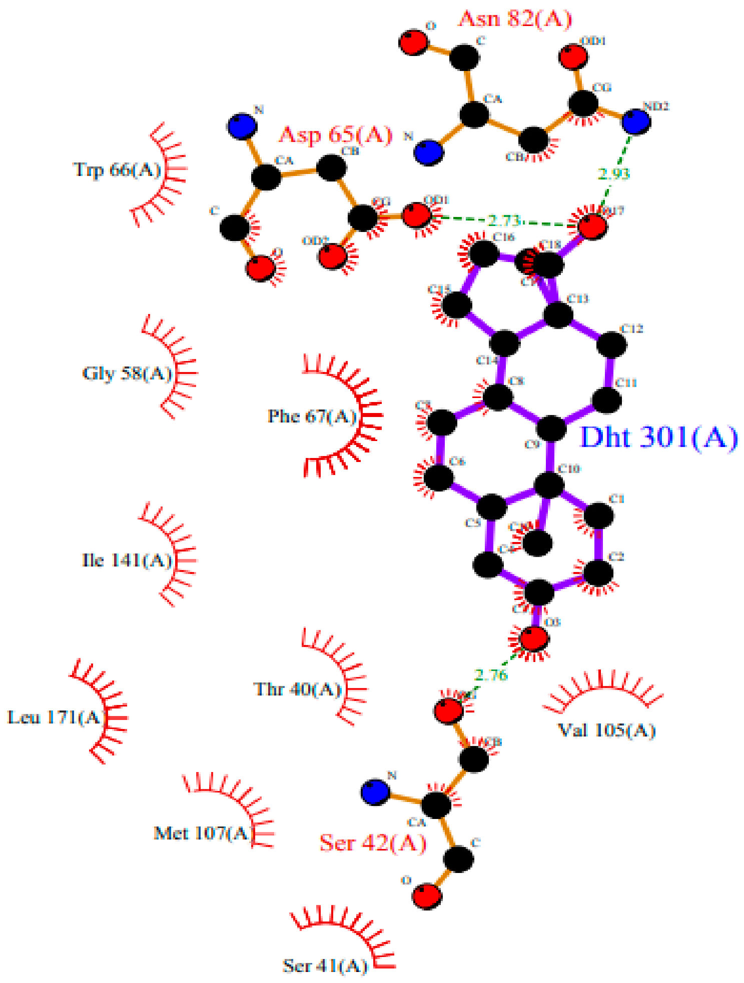

Figure S6. Represents active site residues are SER, ASP and ASN.

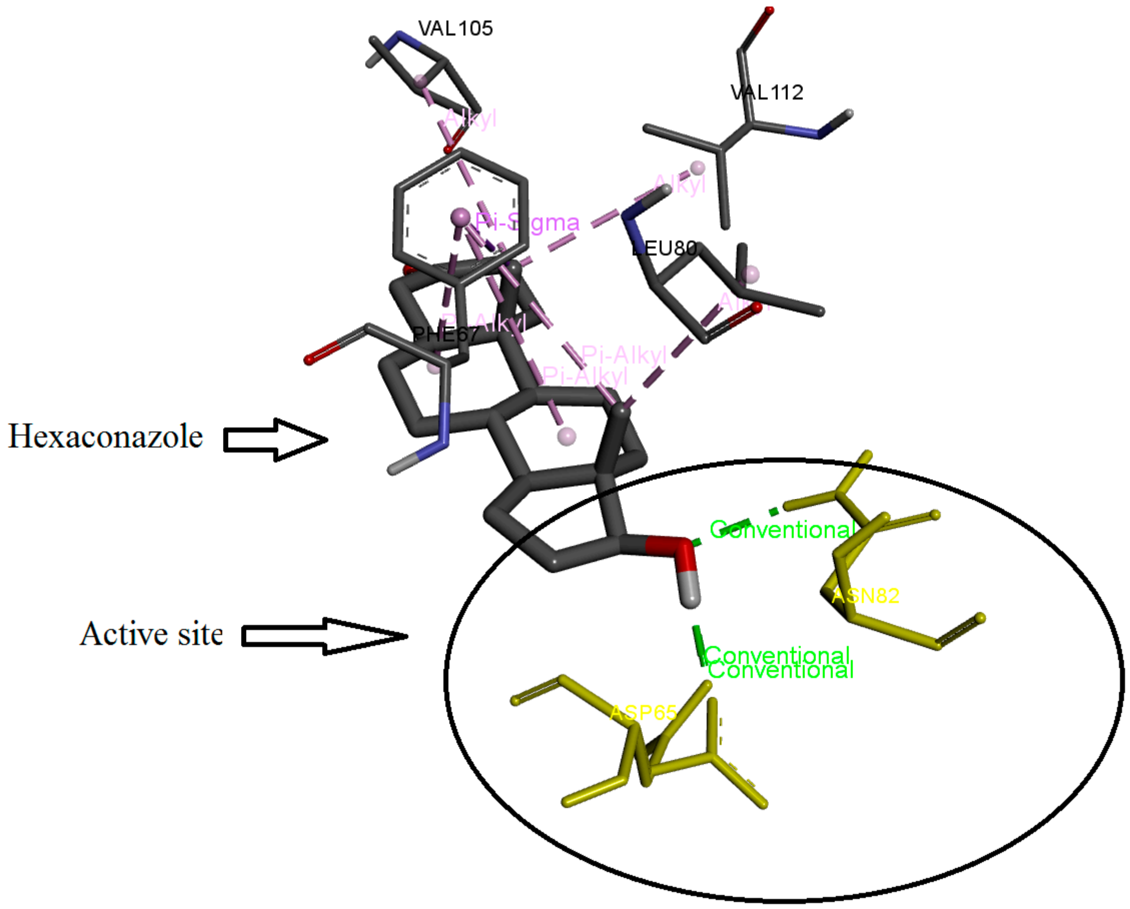

Figure S7. Represents hexaconazole form hydrogen bond with active site residues ASP and ASN.

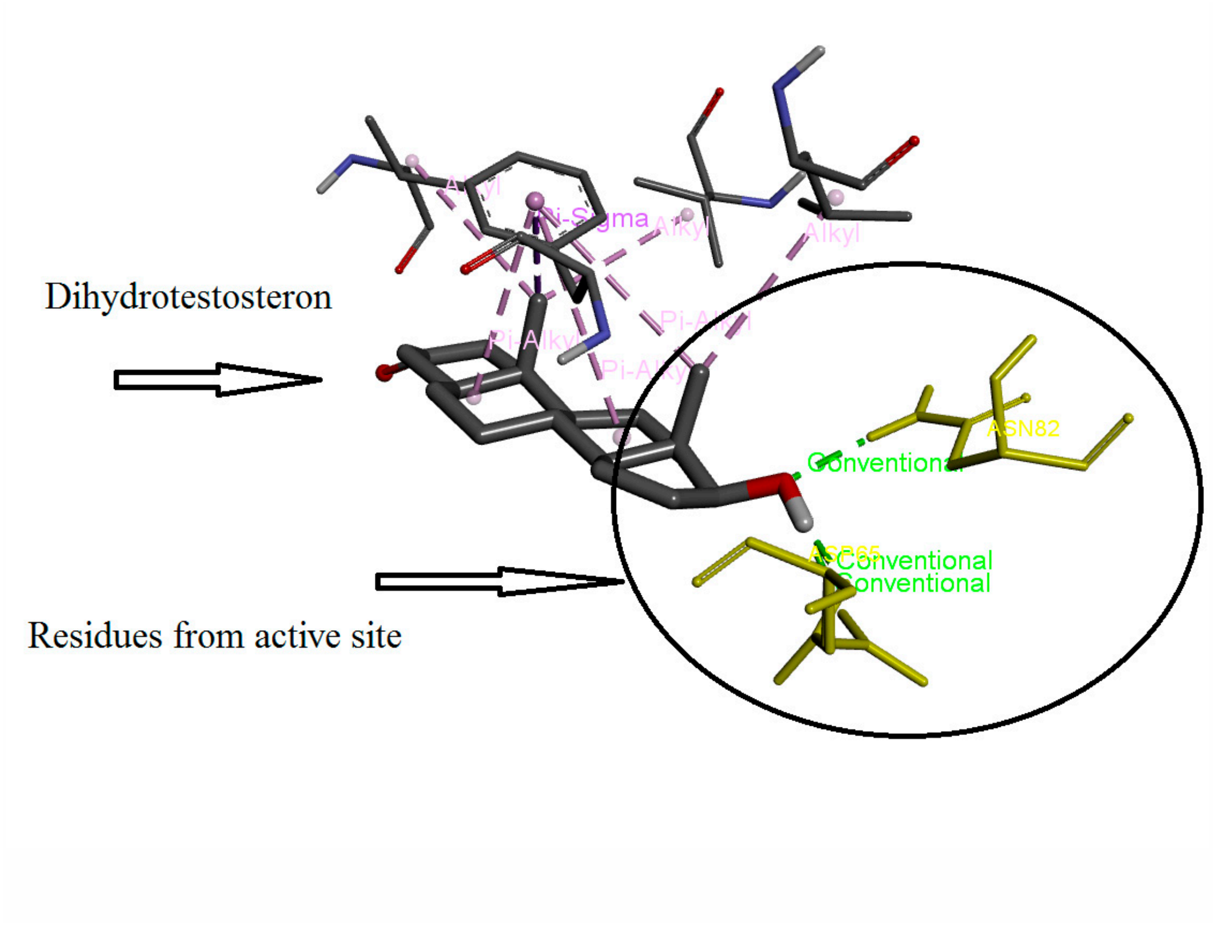

Figure S8. Represents Dihydrotestosterone form hydrogen bond with active site residues ASP and ASN.

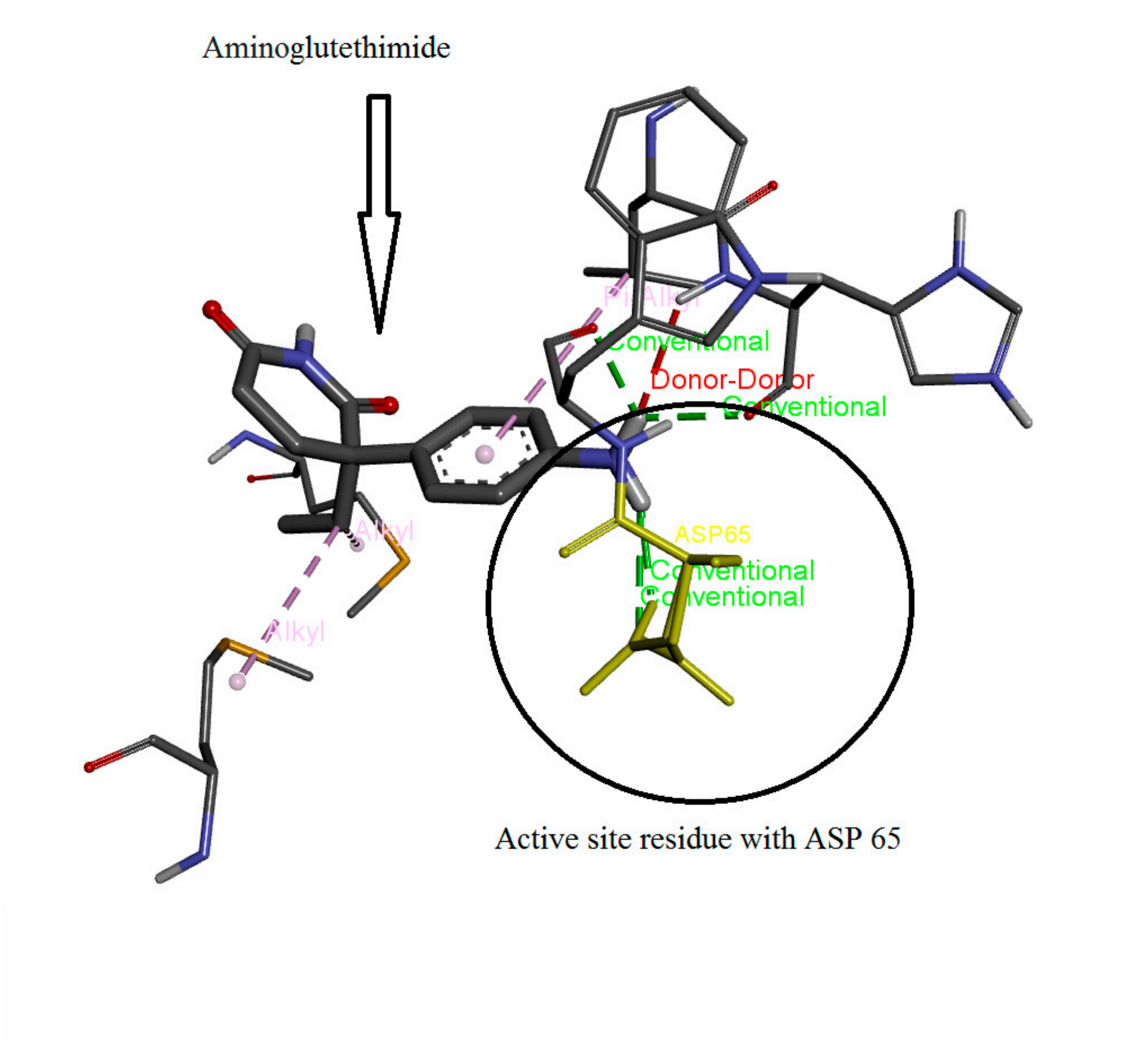

Figure S9. Represents Aminoglutethimide form hydrogen bond with active site residues ASP.

Supplementary **Figure S10**. comparison of hydrogen bond during MD simulation with 10 ns and 30 ns is given below.

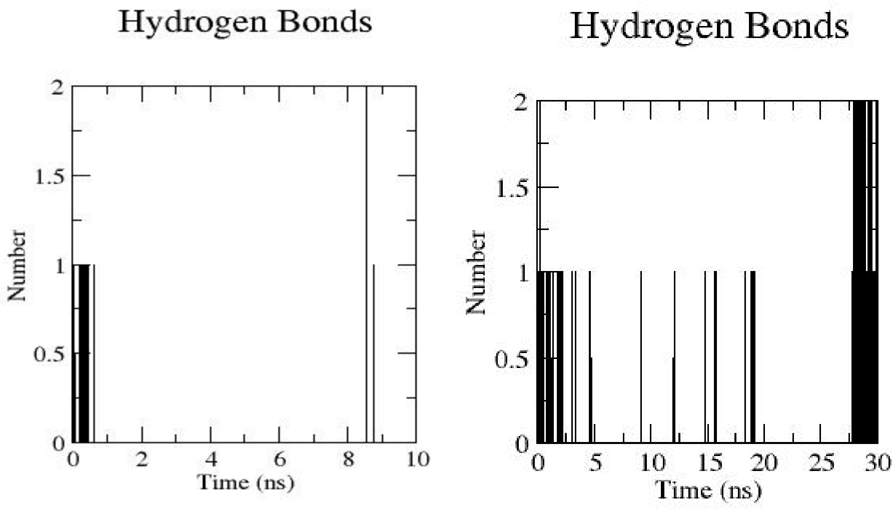

A B

Hexaconazole hydrogen bond formation after 10 ns Figure A and after 30 ns Figure B

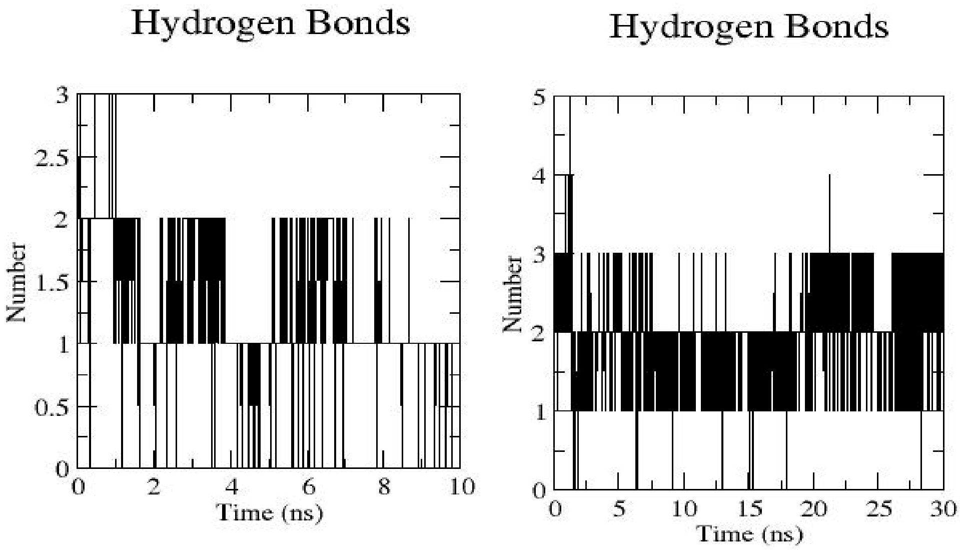

A B

Dihydrotestosterone hydrogen bond formation after 10 ns Figure A and after 30 ns Figure B

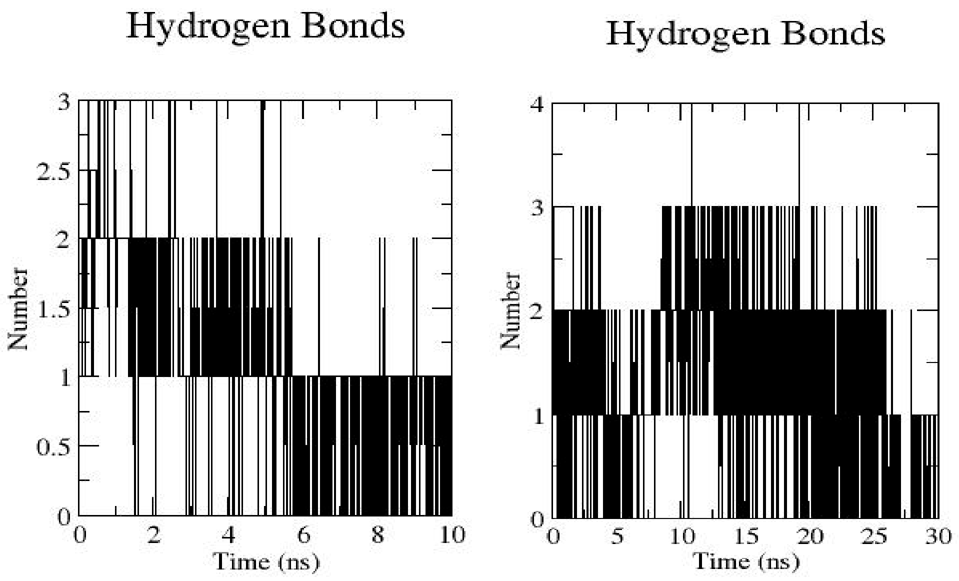

Aminoglutethimide hydrogen bond formation after 10 ns Figure A and after 30 ns Figure B

Image S1

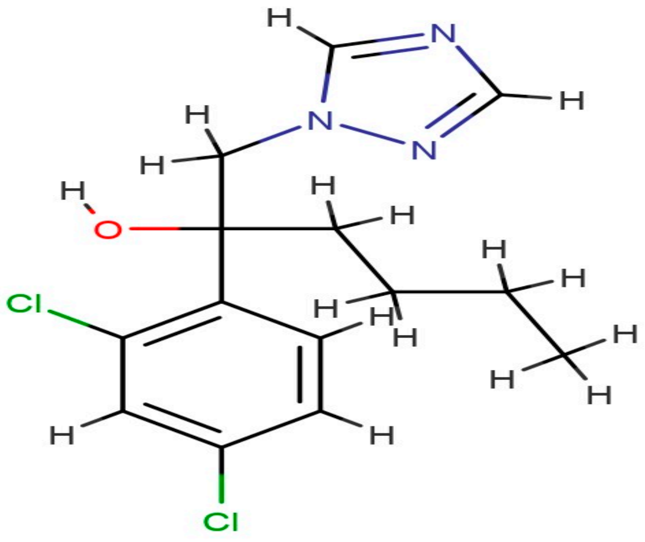

Hexaconazole

2-(2,4-Dichlorophenyl)-1-(1*H*-1,2,4-triazol-1-yl)hexan-2-ol

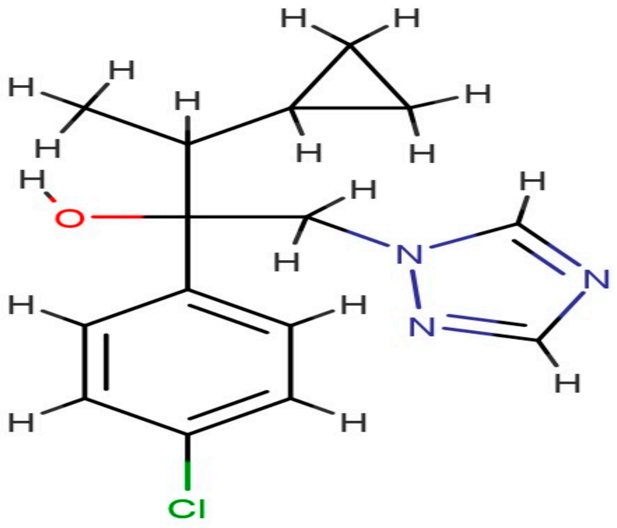

Cypronazole 86132

2-(4-Chlorophenyl)-3-cyclopropyl-1-(1*H*-1,2,4-triazol-1-yl)-2-butanol

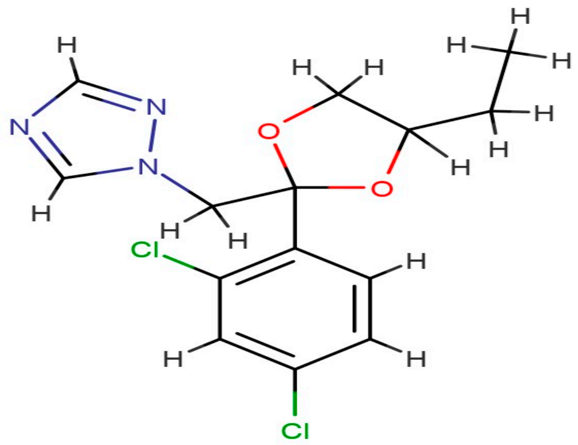

1-[[2-(2,4-dichlorophenyl)-4-ethyl-1,3-dioxolan-2-yl]methyl]-1,2,4-triazole

Etaconazole   91673

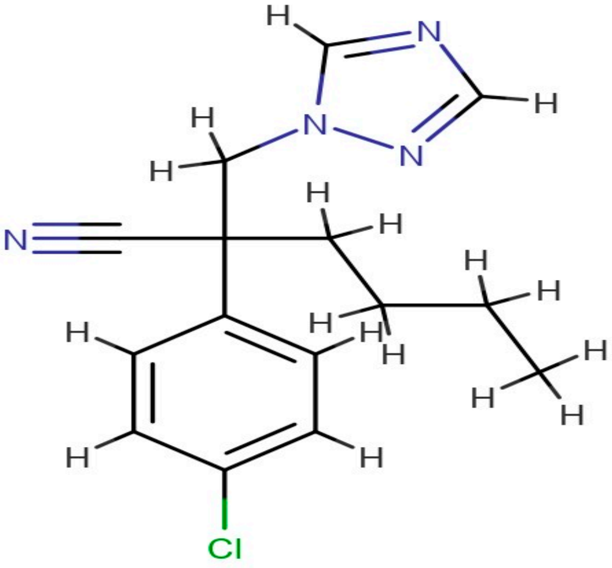

2-(4-Chlorophenyl)-2-(1,2,4-triazol-1-ylmethyl)hexanenitrile

Myclobutanil 6336

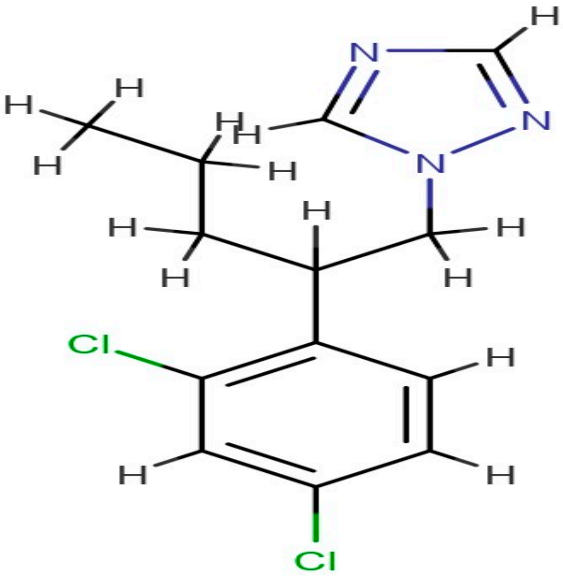

Penconazole 91693

1-[2-(2,4-dichlorophenyl)pentyl]-1,2,4-triazole

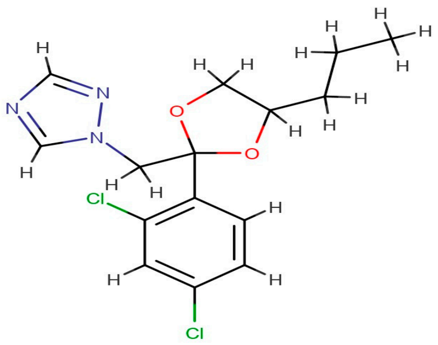

Propiconazole 43234

1-[[2-(2,4-dichlorophenyl)-4-propyl-1,3-dioxolan-2-yl]methyl]-1,2,4-triazole
